# Research on B Cell Algorithm for Learning to Rank Method Based on Parallel Strategy

**DOI:** 10.1371/journal.pone.0157994

**Published:** 2016-08-03

**Authors:** Yuling Tian, Hongxian Zhang

**Affiliations:** College of Computer Science and Technology, Taiyuan University of Technology, Taiyuan, China; Tianjin University, CHINA

## Abstract

For the purposes of information retrieval, users must find highly relevant documents from within a system (and often a quite large one comprised of many individual documents) based on input query. Ranking the documents according to their relevance within the system to meet user needs is a challenging endeavor, and a hot research topic–there already exist several rank-learning methods based on machine learning techniques which can generate ranking functions automatically. This paper proposes a parallel B cell algorithm, RankBCA, for rank learning which utilizes a clonal selection mechanism based on biological immunity. The novel algorithm is compared with traditional rank-learning algorithms through experimentation and shown to outperform the others in respect to accuracy, learning time, and convergence rate; taken together, the experimental results show that the proposed algorithm indeed effectively and rapidly identifies optimal ranking functions.

## Introduction

Rank-learning applications for information retrieval (IR) have garnered increasing research attention in recent years. (The benchmark dataset for testing rank-learning methods is Microsoft LETOR [1].) “Learning to rank” involves the use of machine-learning techniques, as well as other related technologies to learn datasets in order to automatically generate optimal ranking functions; ranking function performance essentially depends on the rank-learning algorithm. Rank-learning is widely used in many applications associated with ranking tasks. For example, Yu J et al. [2] propose a novel ranking model for image retrieval based on the rank-learning framework, in which visual features and click features are simultaneously utilized to obtain the ranking model. Liu B et al. [3] propose a new computational method called ProtDec-LTR for protein remote homology detection, which is able to combine various ranking methods in a supervised manner via using the rank-learning algorithm. The results indicate predictive performance improvement can be achieved by combining different ranking approaches in a supervised manner via using rank-learning. Yang X et al. [4] introduce a learning-to-rank approach to construct software defect prediction models by directly optimizing the ranking performance. They empirically demonstrate that directly optimizing the model performance measure can benefit software defect prediction model construction. Chen J et al. [5] propose a rank-learning based framework for assessing the face image quality, because selecting face images with high quality for recognition is a promising stratagem for improving the system performance of automatic face recognition.

Traditional rank-learning algorithms are dependent on loss function optimization. Because the ranking function is evaluated by certain measures such as mean average precision (MAP) or normalized discounted cumulative gain (NDCG), ideally, the loss function is built through evaluation measures. Numerous algorithms have been proposed previously based on such IR evaluation measures [6], in addition to methods based on evolutionary computation. Genetic programming methodology has been particularly successfully applied to the design of rank-learning algorithms [7–8]. The clonal selection algorithm, which is based on the artificial immune system and immune programming, has also been applied to design rank-learning algorithms [9–10].

The traditional rank-learning algorithm is similar to the traditional machine-learning algorithm, where most optimize the loss function to generate a ranking function with minimum loss through iterations [11]. The loss function itself determines which mathematics principia or machine learning techniques are applied for optimization. For ListWise [12] methods, for example, typical loss functions are based on IR evaluation measures such as MAP, NDCG, or P@n. IR evaluation measures are integrated into loss functions, then the learned result naturally shows favorable evaluation measures. Loss functions based on IR evaluation measures are not smooth, however, and thus cannot be optimized via traditional machine-learning techniques–only upper bound functions or similar functions of the original loss function can be optimized by traditional machine learning techniques.

Traditional rank-learning methods based on loss functions utilize the analyticity properties of the loss function and geometric features of the constraint space to gradually shrink the search space in order to find optimal solutions. As the problem size increases, though, the traditional loss-function-based algorithm is no longer able to obtain the optimal solution within an acceptable timeframe. It is necessary (and urgent, considering the current demand) to establish an intelligent optimization method based on IR evaluation measures that can work sufficiently quickly (i.e., at reduced computation time.)

The B cell algorithm [13] is an immune algorithm based on the clonal selection principle which can start from a set of feasible solutions without any loss function to evolve and facilitate efficient searching, eventually returning global optimal solutions. Previous studies have shown that the B cell algorithm is convergent and requires fewer iterations compared to the hybrid genetic algorithm or clonal selection algorithm without affecting the quality of the solution results [14]. The B cell algorithm has natural parallel characteristics and is very well-suited to multi-CPU parallel computing, which not only allows full use of modern computer hardware resources to accelerate the algorithm’s speed, but also reduces the possibility of local optima and improves the quality of optimal solutions by expanding single populations to multiple populations with rich species diversity.

MapReduce is an easy-to-use and general-purpose parallel programming model that is suitable for analyzing large data sets. The Apache Hadoop gives researchers the possibility of achieving scalable, efficient, and reliable computing performance on Linux clusters. The MapReduce model has been applied to parallel computation with large datasets in the bioinformatics field [15–16], but in the field of learning to rank, the dataset size used for training and testing (LETOR, as mentioned above) is only several dozens of megabytes. The core distributed feature of MapReduce cannot be utilized fully to design parallel rank-learning algorithms, and MapReduce is not suitable at all for iterative training in rank learning. Wang S et al. [17] propose a parallel framework called CCRank for learning to rank based on evolutionary algorithms. The method is based on cooperative coevolution (CC), which is a divide-and-conquer framework that has demonstrated high promise in function optimization for problems with large search space and complex structures. In this study, we applied a simple parallel strategy to the B cell algorithm and found that the resulting parallel B cell algorithm can execute rank-learning tasks effectively on a multi-core processor.

This paper presents a parallel B cell algorithm that was developed to improve the precision and speed of rank-learning tasks. The novel algorithm is a type of coarse-grained, master-slave parallel model: An initial population is generated by the master node and then divided into multiple subpopulations to evolve independently. During the evolution process, each clone pool of every individual crosses over to increase the population diversity and enrich the search space. Parallel computing can speed up the evolution of the entire population so as to obtain the global optimal solution rapidly.

## Rank Learning

Ranking, as discussed above, is the primary issue in IR applications. “Ranking” in this context involves securing a ranking function that can respond to user query to rank documents based on their relevance within the corpus. The ranking problem can be formalized as follows.

Given a query *q*_*i*_ ∈ *Q*, |*Q*| = *m* as well as a set of documents *d*_*i*_ = $\left\{d_{i1},d_{i2},\ldots ,d_{i,n(q_{i})}\right\}$ associated with *q*_*i*_, then the degree of relevance between *q*_*i*_ and *j*-th document *d*_*ij*_ is defined as follows: S1 Equation, where *r*_*i*_ is the degree of relevance, *r*_*n*_≻*r*_*n*−1_≻*r*_*n*−2_≻…≻*r*_1_, ≻ represents preference relations, *x* is a feature vector, *ϕ* is a feature extraction function, and *n*(*q*_*i*_) is the number of documents associated with *q*_*i*_. For a given *q*_*i*_, the evaluation function between *π*_*i*_ and *y*_*i*_ is *E*(*π*_*i*,_ y_*i*_), where *π*_*i*_ is the sequence generated by the descending order of documents associated with *q*_*i*_. For document retrieval, the essence of the ranking function is to compute the relevance between the document and the query, then to rank documents by relevance–accordingly, the ranking function usually refers to the relevance calculation function.

Generating a ranking function includes three main factors: First, the representation of the degree of relevance; second, the relevance calculation method; and third, the features of the query-document pair. Different representations of relevance and calculation methods can produce entirely different ranking functions. Most of the traditional ranking functions are based on the “word bag” pattern, that is, the term frequency (TF) and inverse document frequency (IDF) attributes serve to calculate relevance. For example, the vector space model represents relevance degree as the angle between the two vectors in a vector space, where the calculation method is the inner product of the vectors. The probability model represents the relevance degree as the probability a document is relevant with a given query, where calculation is built on the conditional probability model and independent event probability model.

Traditional ranking model design generally takes place in the following steps.

1. Extract features from the query-document pairs (e.g., TF, IDF).
2. Specify the representation of relevance between a query and a document.
3. In accordance with the degree of relevance, use the known relevance calculation method to combine features and obtain the initial ranking function.
4. Adjust the parameters in the ranking function to make the ranking function utile in practice.

The traditional ranking function is simple and easy to calculate, but recent advancements in IR (especially modern search engines,) have left simple ranking functions unable to adapt to highly complex and dynamic user needs. Search engines receive a wealth of user feedback and logs on a daily basis, and new features cannot automatically be added to traditional ranking functions, which makes them difficult to improve as necessary.

Rank learning is a machine-learning technique employed to automatically obtain optimal ranking functions during IR. Machine learning has four main components: input space, output space, hypothesis, and machine-learning algorithms. The historical information supplied for a machine to “learn” commonly refers to training sets (which may include manual labels input for supervision.)

Applying rank-learning techniques to automatically create a ranking function needs the following steps.

1. Prepare training collection *D* = ${\left\{\left(q_{i},d_{i},y_{i}\right)\right\}}_{i=1}^{m}$: The training set contains a collection of queries, a set of documents related to each query, and a relevance judgment for each query-document pair.
2. Design the rank-learning algorithm.
3. Apply the rank-learning algorithm to the training set *D* and automatically generate the optimal ranking function.
4. Evaluate the generated ranking function and compare it against the existing ranking functions to decide whether the ranking function performs effectively in practice.
5. Apply the favorable ranking function to unseen data sets, where given a set of queries and related documents, the documents are ranked by relevance and the more relevant documents are placed into upper positions.

Among the above five steps, the learning algorithm design step is the key to the entire process. Algorithm design depends on the hypothesis space, the form of the training set, and the loss function. The B cell algorithm, as mentioned above, is an immune algorithm based on the clonal selection mechanism which conducts evolution on the initial solution space to search a group of optimal solutions. It is an effective, “natural” machine-learning algorithm that features relatively rapid search speed by representing the ranking function as the “antibody” in the population, then evaluates it on the specified dataset through an IR evaluation function to guide the learning process into the optimal solution space.

## B Cell Algorithm

The B cell algorithm (BCA) is an immune-inspired algorithm which includes a distinguished clonal selection process and mutation mechanism. BCA can be applied to various types of optimization problems and shows better performance than the hybrid genetic algorithm or clonal selection algorithm. An important feature of BCA is its particular mutation operator, continuous region hypermutation, the biological motivation for which is as follows: When a mutation occurs on the B-cell receptors, the system focuses on determining complementary, small regions on the receptor, i.e., sites that are primarily responsible for detecting and binding to their targets. This process basically forms a highly focused search. BCA accordingly forms an interesting contrast with the method employed by CLONALG, whereby although multiple mutations take place, they are uniformly distributed across the vector rather than being targeted at a contiguous region. The contiguous mutation operator, rather than selecting multiple random sites for mutation, chooses a random site (or hotspot) within the vector along with a random length; the vector is then subjected to mutation from the hotspot onward until the length of the contiguous region has been reached. The other most notable feature of BCA is its independence during the antibody evolution process. The father antibody produces a child clone pool in each iteration, then the child clone pool expands the search space through mutation. Finally, the father antibody is replaced by its most fit child to realize the population evolution.

The BCA framework is as follows.

1. Initialize a random individual (antibody) population *P*.
2. For individual *v* ∈ *P*, apply a fitness function *g*(*v*) to *v*.
3. Duplicate *v* ∈ *P* and place the clones in clone pool *C*.
4. Apply the mutation operator to all the individuals in *C* to get clone pool *C′*.
5. Compute the fitness of each individual *v′* ∈ *C′*; if *g*(*v*′) > *g*(*v*), then replace *v* with *v′*.
6. Loop from Step 2 to Step 5 until the stop condition is met.

## Parallel B Cell Algorithm

**Fig 1:**
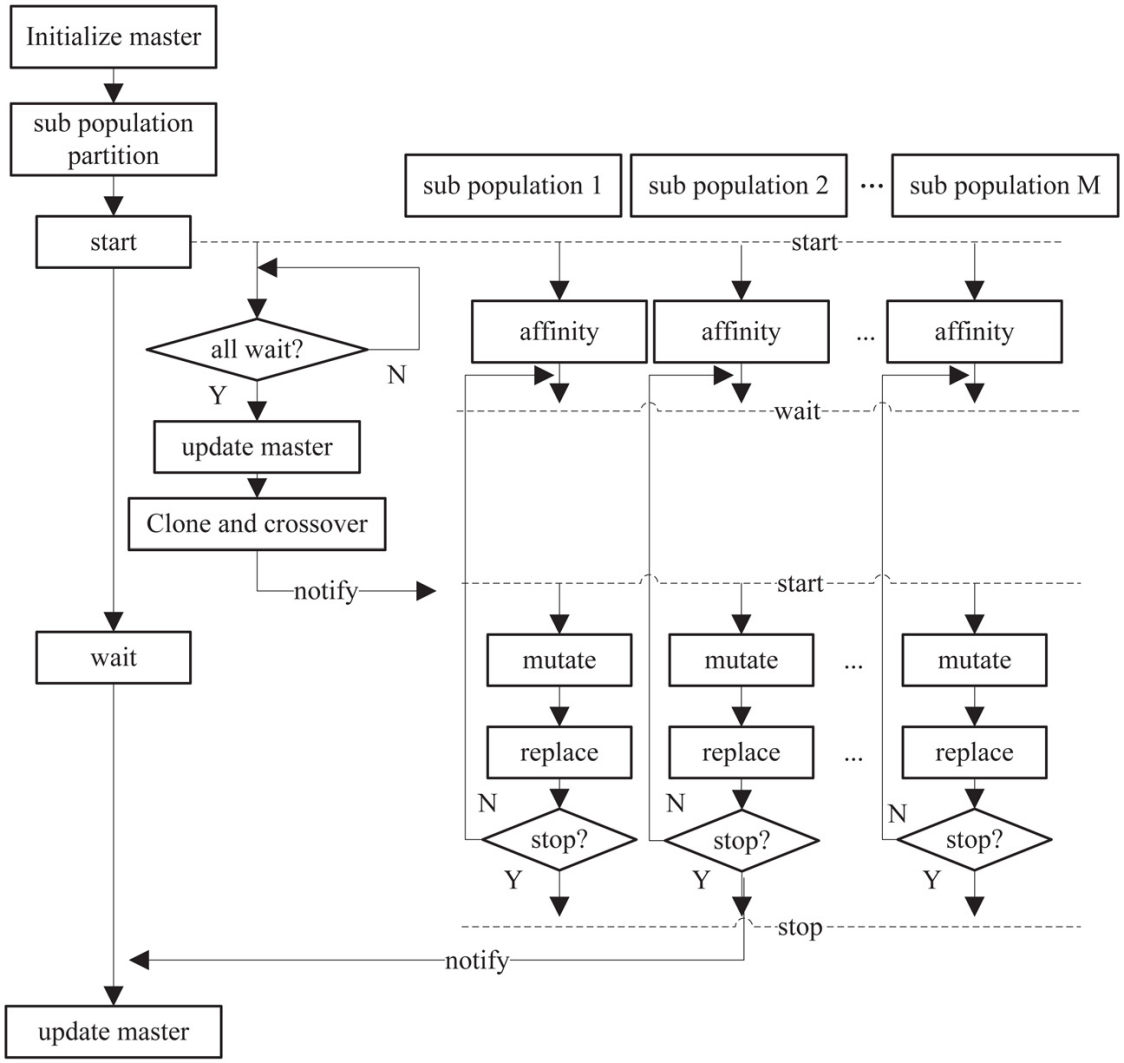
Parallel model of B cell algorithm.

There are two important reasons to parallelize the BCA: The first is to increase computational efficiency by using multiple cups to conduct the same learning task, and the second is to research the parallel model of the BCA to ensure its original arithmetic features are maintained, allowing it to be applied to several machine-learning fields. The parallel model of the BCA is shown in Fig 1.

Multiple execution individuals (such as threads) complete the same task in a parallel manner collaboratively in the proposed algorithm. Each thread completes the same amount of work through the serial method. Apart from speeding up the learning process, the parallel model also introduces a crossover operator to ensure rich diversity of each clone pool. Compared to the original serial algorithm, the proposed algorithm expands the search space of the whole population after each iteration to speed up the convergence rate.

Parallel BCA adopts the following step-wise process.

(1) The master node reads the training dataset as the antigen set *G* = (*Ag*_1_, *Ag*_2_, …, *Ag*_*g*_). The initial antibody population size is defined as *n*, clone pool size as *n*_*c*_, antibody encoding length as *l*, processor size as *M*, and antibody gene set as *B* = {*ab*_1,_ *ab*_2_, …, *ab*_*m*_}.

The antibody space is defined as:

$$I^{l}=\left\{Ab:Ab=(ab_{1},ab_{2},\ldots ,ab_{l}),ab_{k}\in B,1\leq k\leq l\right\}$$

Then the antibody population space is defined as:

$$P^{n}=\left\{P:P=(Ab_{1},Ab_{2},\ldots ,Ab_{n}),Ab_{k}\in I^{l},1\leq k\leq n\right\}$$

The antibody population after the *k*-th iteration is P(*k*) = {*Ab*_1_(*k*), *Ab*_2_(*k*), …, *Ab*_*n*_(*k*)} ∈ *P*^*n*^, then *k* = 0 is initialized and iteration number is *Gen*. The initial population, i.e., master population, is *P*(0).

(2) An equal division of population *P*(0) is defined, that is, $P(0)=\overset{M}{\underset{j=1}{\cup }}Sub_{j}$ and the subpopulation size U=|Subj|=nM,1≤j≤M.

(3) *Sub*_1_, …, *Sub*_*M*_ to *M* processors are assigned; each subpopulation *Sub*_*j*_ processes in parallel.

The affinity of antibody *Ab* ∈ *Sub*_*j*_ is defined as:

$$AVAF(Ab,G)=\frac{\sum_{i=1}^{g}AF(Ab,Ag_{i})}{g},$$

where *AF* is the antibody-antigen affinity function and *AVAF* is the average affinity on the antigen set.

(4) Antibody population *P*(*k*) is cloned, and clone size is *n*_*c*_. The clone pool of antibody *Ab*_*i*_(*k*) ∈ *P*(*k*) is *Pool*(*Ab*_*i*_(*k*)), where 1 ≤ *i* ≤ *n*.

(5) The antibodies of clone pools cross over; the basic principle of the crossover is to maximize antibody diversity in the clone pools. The crossover operation includes “entire cross” and “partial cross”, and is defined as follows.

In one iteration, put master population into a list *L*, where *L* = (*Ab*_1_, *Ab*_2_, …, *Ab*_*n*_). Divide *L* into *group* groups:

$$group=\left\{\begin{array}{cc} 1 & if\;n<=n\\ \begin{array}{l} n/n_{c}\\ n/n_{c}+1 \end{array} & \begin{array}{l} n>n_{c}\;and\;n\%n_{c}=0\\ n>n_{c}\;and\;n\%n_{c}!=0 \end{array} \end{array}\right\}$$

Then *L* = (*L*_1_, *L*_2_, …, *L*_*group*_).

If *n*> = *n*_*c*_ and *n*%*n*_*c*_ = 0, and *L*_*i*_ ∈ *L*, |*L*_*i*_| = *n*_*c*_, then $L_{i}=(Ab_{(i-1)n_{c}+1},Ab_{(i-1)n_{c}+2},\ldots ,Ab_{(i-1)n_{c}+n_{c}})$, where 1 ≤ *i* ≤ *group*,$Ab_{(i-1)n_{c}+j}\in L,1\leq j\leq n_{c}$. Antibody $Ab_{(i-1)n_{c}+j}$ forms clone pool $Pool_{ij}=(Ab_{ij1},Ab_{ij2},\ldots ,Ab_{ijn_{c}})$, and operation *swap*(*Ab*_1_, *Ab*_2_) is defined as the exchange between antibody *Ab*_1_ and antibody *Ab*_2_. Then the crossover process of clone pool in *L*_*i*_ is:

*for*(*j* = 1; *j*<*n*_*c*_; *j*++)

 *for*(*s* = 1, *k* = *j*; *k*<*n*_*c*_; *k*++, *s*++)

  *swap*(*Ab*_*ijk*_, *Ab*_*i*(*j*+*s*)*j*_)

Because each antibody in a clone pool is different after crossover, this kind of crossover is called “entire cross”.

If *n* > *n*_*c*_ and *n%n*_*c*_ ≠ 0, and *L*_*group*_ ∈ *L*, |*L*_*group*_|≠*n*_*c*_, then the crossover in *L*_*group*_ is as follows:

*for*(*j* = 1; *j*<*n*%*n*_*c*_; *j*++)

 *for*(*s* = 1, *k* = *j*; *k*<*n*%*n*_*c*_; *k*++, *s*++)

  *swap*(*Ab*_*ijk*_, *Ab*_*i*(*j*+*s*)*j*_)

Because there still exists the same antibody in *L*_*group*_ after crossover, this kind of crossover is called “partial cross”.

If *n* < *n*_*c*_, *group* = 1, then the crossover in *L*_*group*_ is as follows:

*for*(*j* = 1; *j*<*n*; *j*++)

 *for*(*s* = 1, *k* = *j*; *k*<*n*; *k*++, *s*++)

  *swap*(*Ab*_*ijk*_, *Ab*_*i*(*j*+*s*)*j*_)

Because there still exists the same antibody in *L*_*group*_ after crossover, this kind of crossover is also called “partial cross”.

(6) Each processor conducts mutations on antibodies in the clone pool in parallel. When the *k*-th iteration is reached, clone pool *Pool*(*Ab*_*i*_(*k*)) is changed to *Pool*′(*Ab*_*i*_(*k*)) after mutation. The antibody in *Pool*'(*Ab*_*i*_(*k*)) is represented as *PAb*′_*j*_ ∈ *Pool*′(*Ab*_*i*_(*k*)), 1≤*i*≤*n*, 1≤*j*≤*n*_*c*_.

(7) Each processor chooses the best antibody in the mutated clone pool to replace its father antibody in parallel. For antibody *Ab*_*i*_(*k*) in the master population, the replacement process is as follows:

*for*(*j* = 1; *j*< = *n*_*c*_; *j*++)

 *if*(*affinity*(*PAb*′_*j*_, *G*)> *affinity*(*Ab*_*i*_(*k*), *G*))

  *replace*(*Ab*_*i*_(*k*), *PAb*′_*j*_)

where *replace*(*Ab*_*i*_(*k*), *PAb*′*j*) represents replacing *Ab*_*i*_(*k*) with *PAb*′*j*.

(8) Loop Form (3) to (7) until the iteration reaches *Gen*.

(9) The master node collects the best antibodies from each subpopulation to form the final optimal solutions $P(Gen)=\overset{M}{\underset{j=1}{\cup }}Sub_{j}$.

## RankBCA: A Rank-learning Algorithm Based on Parallel BCA

RankBCA is parallel BCA application that can be used to solve ranking problems in IR. It treats training example (*q*_*i*_, *d*_*i*_, *y*_*i*_) as the antigen, ranking function *r*(*x*) as the antibody, and evaluation measure MAP as the affinity function; the training process is in query units. The ranking function computes a relevance score for each document-query pair and evaluates the performance. The MAP, which reflects how well a ranking function performs on a training dataset, is computed after all the queries have been evaluated. After antibodies in the clone pool have been mutated, the MAP of each ranking function is computed again. If the child’s MAP is larger than its father’s, then the father is replaced by the child. This process refreshes the ranking functions in the initial antibody repository to obtain a collection of optimal ranking functions. In order to secure the best ranking function from the collection, each ranking function computes the MAP on the training dataset and the MAP on the validation dataset, then computes the average MAP between them; the ranking function with the largest average MAP is the final output.

There are three kinds of nodes involved in parallel learning: Master nodes, slave nodes, and cross nodes. The master node is responsible for starting the learning and tail-in work; the slave node is responsible for subpopulation evolution; and the cross node is responsible for cloning and crossover. The RankBCA process is outlined below.

(1) initialization

The master node randomly generates an antibody repository (master population) accompanied by ranking functions. The master population is divided into several subpopulations, each of which is assigned to a single slave node. The slave nodes and cross node are then started and the LETOR training and validation datasets are initialized. The training dataset is used for training, and the validation dataset is used to select the optimal ranking function.

(2) training in parallel

The slave node is responsible for training each subpopulation; each slave node executes the task in parallel and enters a wait state after finishing the replacement. The cross node conducts cloning and crossover on the master population, then notifies all slave nodes after finishing crossover; once the master node is notified, it proceeds to update the master population while the slave nodes finish learning.

(3) choose the optimal ranking function

The master node selects the optimal ranking function from the master population after training. As discussed above, the average MAP is computed between the MAP on the training dataset and that on the validation dataset for each ranking function in the master population, then outputs the ranking function with the largest average MAP value.

### Antibody and Antigen

The antigen, antibody, and affinity are the three components of BCA. Using BCA to solve rank-learning problems involves identifying the correspondence between immune components and rank-learning components. During immune modeling, each antigen expresses a problem that is typically represented as a mapping of inputs and outputs. Each antibody candidate in the antibody repository expresses a solution and is randomly generated by a gene pool, and the affinity expresses fitness between the antibody (candidate solution) and the antigen (problem) [18].

Rank learning automatically generates a ranking function that can calculate the relevance score between a query and document. Accordingly, the antigen can be represented as a mapping of an input and output (*q*_*i*_, *d*_*ij*_, *y*_*ij*_). The IR evaluation measure is query-based, so the antigen itself must likewise be query-based: The antigen is defined as (*q*_*i*_, *d*_*i*_, *y*_*i*_), and the antibody is a candidate formed by ranking function *r*(*x*). In the LETOR dataset, a query-document pair is represented as a real feature vector *x* = *ϕ*(*q*_*i*_, *d*_*ij*_), i.e., feature values that are a part of the gene pool. The antigen repository represents the entire training set *D* = ${\left\{\left(q_{i},d_{i},y_{i}\right)\right\}}_{i=1}^{m}$, which is also a collection of multiple antigens.

The affinity, which expresses the goodness of any ranking function, is defined as IR evaluation measure *E*(*π*_*i*_, *y*_*i*_). The correspondence between immune components and rank-learning components is summarized in Table 1.

**Table 1:** Correspondence between immune components and rank-learning components.

Immune Components	Rank-learning Components
Antigen *Ag*_*i*_	List of documents and labels (*q*_*i*_, *d*_*i*_, *y*_*i*_)
Antibody *Ab*_*i*_	Ranking function *r*(*x*)_*i*_
Training repository $R={\left\{Ag_{i}\right\}}_{i=1}^{m}$	Training set $D={\left\{\left(q_{i},d_{i},y_{i}\right)\right\}}_{i=1}^{m}$
Validation repository $V={\left\{Ag_{i}\right\}}_{i=1}^{k}$	Validation set $VD={\left\{\left(q_{i},d_{i},y_{i}\right)\right\}}_{i=1}^{k}$
Affinity *AF*(*Ab*_*i*_, *Ag*_*j*_)	IR evaluation measure *E*(*π*_*j*_, *y*_*j*_)

### Gene Pool and Antibody Tree

The “gene pool” defines the antibody structure space. During rank learning, the antibody represents a function computing a score with a real vector; each dimension in the real vector is represented as a variable in a function. A ranking function not only contains variables, however, but also operators and constants, i.e., ranking function *r*(*x*) = (3*x_1_ + 4*x_2_)/(2−*x*_3_), in which *x* = (*x*_1_, *x*_2_, *x*_3_) is a three-dimensional real vector and operators are the elements in {+, *,/−}; constants are 2, 3, and 4. In short, the gene pool contains a feature set, operator set, and constant set.

The gene pool is defined as *I* = {*F*, *O*, *C*}, where *F* is the feature set, *O* is the operator set, and *C* is the constant set. The gene pool of RankBCA is defined as follows:

$$F=\left\{f_{i}|f_{i}\in x,i\in Z\wedge i\in [1,dimension(x)]\right\}$$

O={+,−,*,/}

$$C=\left\{\begin{array}{l} 0.\text{1},0.\text{2},0.\text{3},0.\text{4},0.\text{5},0.\text{6},0.\text{7},0.\text{8},0.\text{9},\text{1}.0,\\ \text{2}.0,\text{3}.0,\text{4}.0,\text{5}.0,\text{6}.0,\text{7}.0,\text{8}.0,\text{9}.0,\text{1}0.0 \end{array}\right\}$$

where *dimension*(*x*) is the dimension of the feature vector *x*.

**Fig 2:**
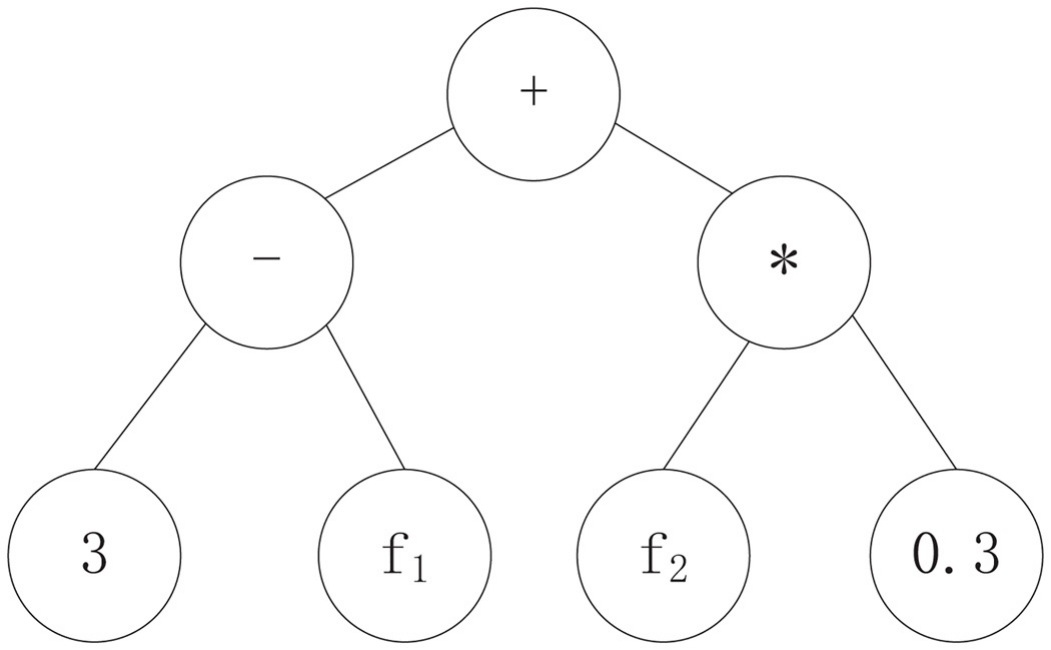
Antibody tree example.

An antibody is built of the three distinct components in the gene pool. The initial antibody contains all of the features, then operators and constants are selected at random. The features are selected at random during antibody evaluation. The choice of antibody data structure has a significant effect on the algorithm’s running efficiency and portability. The traditional methodology is built on the tree and s-expression structure, which previous scholars have established via stack-based architecture [18]. Tree representation is more easily understood and calculated, however, which is why we adopted it here. The operator is an internal node of the antibody, and leaf nodes represent constants and features. An antibody is a candidate ranking function, the function expression of which is generated by inorder traversing the tree. Accordingly, the ranking function does not need to cover all available features. As shown in Fig 2, the ranking function is (3 − *f*_1_) + (*f*_2_*0.3) after inorder traversing the tree.

To construct the antibody tree, an antibody is represented as a full binary tree structure and the nodes in the tree are divided into three types: feature nodes, operator nodes and constant nodes, respectively corresponding to feature set *F*, operator set *O*, and constant set *C* in the gene pool. The size of each node is determined by the height of the tree, and the number of nodes is (2^*H*^−1) given a tree with height *H*. Each node in the antibody tree has a unique serial number which increases from top to bottom and left to right in the tree. The number of nodes ranges within *ID* = {*id*|*id* ∈ [1,2^*H*^−1] ^ *id* ∈ *Z*}.

Again, the tree's height determines the number of leaf nodes in the antibody tree and the number of leaf nodes is 2^(*H*−1)^ given an antibody tree with height *H*. To ensure that the number of leaf nodes in the experiment can override all the different features, it is necessary to calculate S2 Equation, where |*F*| depends on the dataset, (|*F*| = 45 in the OHSUMED dataset, and |*F*| = 46 in the MQ2007 and MQ2008.) In our experiment, *H* was set 7 to meet S2 Equation. The algorithm’s time complexity increases as *H* increases, so *H* is generally kept below 10.

### Preordered Antibody Encoding

In order to directly express antibodies, they must be encoded in a linear sequence. Each antibody has a unique encoding sequence which the sequential numbers in the tree are utilized to encode, so the antibody encoding sequence is based on the antibody tree. Contiguous regions in the antibody tree must be in accordance with the contiguous regions of the encoding sequence. Through observation, using the antibody tree shown in Fig 2 as an example, there are the following three kinds of contiguous regions in the tree.

1. Nodes are continuous if they are in a subtree, such as <2, 4, 5>.
2. Nodes are continuous if they are located around several subtrees, such as <5, 3, 6, 7>.
3. Nodes are continuous if they are connected directly by layers, such as <1, 2, 4>.

**Fig 3:**
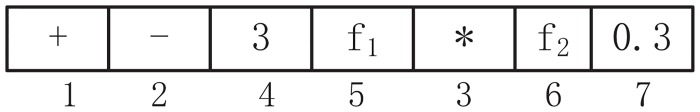
Preordered antibody encoding.

A preorder encoding sequence is applied to encoding the antibody tree in order to maintain consistency between the antibody tree and encoding sequence. Given an antibody tree with height *H*, each element in the encoding sequence is uniquely identified by its sequential number in the antibody tree; the sequential number collection of the elements is *ID* = {*id*|*id* ∈ [1,2^*H*^−1] ^ *id* ∈ *Z*}. An encoding sequence with length 2^*H*^−1 is acquired from a preordered traversal of the antibody tree, then the numbered encoding sequence is *seq* = < 1, 2, 4, …, 2^(*H*−1)^, 2^(*H*−1)^+1, …, 3, …2^*H*^−1>. This final sequence is the preordered encoding of the antibody (Fig 3).

An array is utilized to store the preordered encoding of the antibody. Each element in the sequence is a reference to the node in the antibody tree, a feature which lends the following benefits:

1. It saves memory space. The array elements only store the references to antibodies instead of deep copies of the nodes, so the elements do not contain any additional data.
2. It speeds up mutation. Mutations occur in the linear encoding sequence instead of the antibody trees without traversing the antibody tree.

### Initialization

In RankBCA, *N* antibodies are randomly generated to randomly generate *N* antibody trees. Each node has two important properties: The node sequential number *id* ∈ *ID* and the value of the node *value* ∈ (*F* ∪ *O* ∪ *C*). Elements of type *O* float for the sake of unified computing. A boolean variable *isFeatrue* ∈ {*true*, *false*} represents whether a node is a feature node in the tree, and an integer variable *featrueId* ∈ [1,|*F*|] ^ *featrueId* ∈ *Z* represents the feature identifier of the feature node. The internal nodes and leaf nodes are distinguished to determine whether the left subtree of the node is empty, so there is no need to set additional properties to distinguish internal nodes and leaf nodes. The antibody tree is constructed in three stages: The first stage is to construct the internal nodes in the tree, which are randomly chosen in *O* to generate a full binary tree with height (*H*−1); the second stage is to construct leaf nodes, where |*F*| feature nodes are created (*isFeatrue* is set to true,) and the remaining 2^(*H*−1)^ − |*F*| leaf nodes are randomly selected from *C*; and the third stage is to randomly mount the leaf nodes to the inner nodes. The antibody tree is then traversed by the preordered sequence and the reference node stored into an array, then the final array expresses the preordered encoding of the antibody.

*R* is built on *D* and *V* is built on *VD*. The construction method splits the dataset *D* and *VD* by query, then all the documents associated with the query are constructed to an antigen until all the queries are processed.

### Calculating the Affinity

The affinity between antibody *Ab*_*i*_ ∈ *P* and antigen *Ag*_*j*_ ∈ *R* is defined as follows: S3 Equation. The performance of the antibody is measured by the average affinity of antigen repository *R*. Therefore, evaluation measure *E*(*π*_*j*_, *y*_*j*_) is generally set to MAP to measure the average performance on the test dataset. The average affinity which an antibody *Ab*_*i*_ performs on antigen repository *R* is defined as follows: S4 Equation.

### Antibody Cloning

After an antibody *Ab*_*i*_ ∈ *P* is evaluated by the affinity function, the antibody is cloned to produce a clone pool *C*_*i*_. Clone factor is *β* > 0, and clone size *N*_*c*_ is defined as follows: S5 Equation.

Every antibody is independent and has an independent clone pool *C*_*i*_ in RankBCA, and all the mutations occur in *C*_*i*_.

### Mutation Principles and Mutation Operator

Mutation operation includes both mutation principles and the mutation operator. An antibody tree is presented as a ranking function, where internal nodes can only be the operators and leaf nodes can only be constants or features. Node mutation depends on the type of the node. Mutation principles are defined as follows.

1. The operator node mutates to an operator node in *O* randomly.
2. The constant node mutates to a constant node in *C* or a feature node in *F* randomly.
3. The feature node mutates to a constant node in *C* or a feature node in *F* randomly.

The mutation operator defines a set of mutation behaviors, i.e., contiguous region mutation on the antibody coding sequence; the contiguous region mutation chooses a contiguous region on the antibody coding sequence. The direction of mutation in the original BCA is singular, but the mutation operator in the proposed algorithm works in two directions to reduce the effects of mutation in the right subtree, which ensures that mutations are not only continuous but also that distribution is balanced in the antibody tree. The continuous region mutation operator is defined as follows.

1. A location is randomly selected in the encoding sequence *p* ∈ [1,2^*H*^−1] ^ *p* ∈ *Z* and defined as a “hotspot”.
2. Mutation direction *d* ∈ {−1,1} is chosen randomly, where -1 is left and 1 is right.
3. If *d* = 1, then length *l* ∈ [1,2^*H*^−*p*] ^ *l* ∈ *Z* is chosen randomly and mutated from *p* to the right direction until mutated length *l* (including *p*);
  *else* length *l* ∈ [1, *p*] ^ *l* ∈ *Z* is randomly chosen and mutated from *p* to the left direction until mutated length *l* (including *p*).

**Fig 4:**
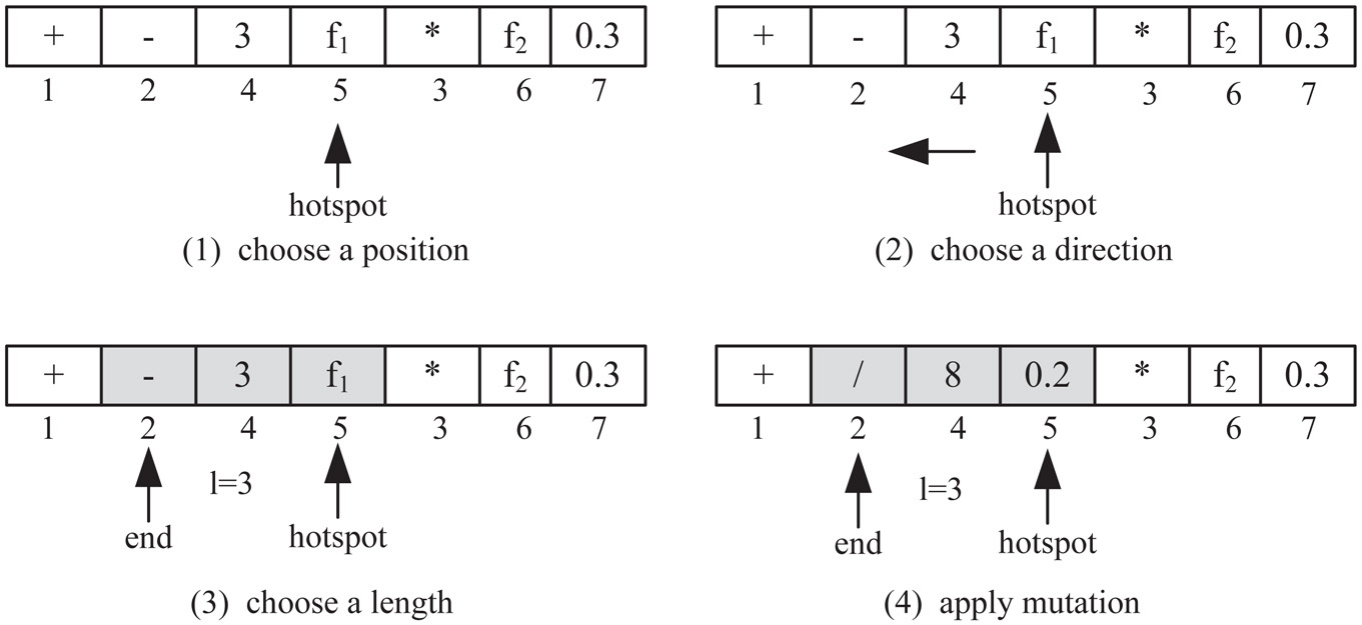
Contiguous region mutation.

For ranking function (3 − *f*_1_) + (*f*_2_*0.3), the mutation process is as shown in Fig 4.

The mutation process in Fig 4 takes place through the following steps:

1. The hotspot is randomly chosen as 5.
2. The mutation direction randomly chosen is left.
3. The mutation length randomly chosen is 3.
4. Nodes are mutated at position 2, 4, 5 according to the mutation principles above; “-” is mutated to “/”, “3” is mutated to “8”, and “f1” is mutated to “0.2” at random.

The mutated ranking function is (8/0.2) + (*f*_2_*0.3).

### Selecting the Optimal Antibody

The master node merges all the subpopulations to update the master population after all the subpopulations have evolved. The master population *P* then has the optimal candidates and the optimal antibody *Ab*_*best*_ ∈ *P* is defined as follows: S6 Equation, where validation antigen repository *V* is employed to verify the algorithm’s performance on new data and to evaluate the generalization of the ranking function. The larger the *AVAF*(*Ab*_*i*_, *V*) value, the better *Ab*_*i*_ performs on new data.

### Detailed Description of RankBCA

As discussed above, the RankBCA algorithm includes three sections respectively represented by the master node, slave node, and cross node. The master node initializes the master population, then starts all the slave nodes and cross nodes and waits for the end of all slave nodes, at which point it merges all the subpopulations on each slave node and selects the optimal ranking function; the algorithm is then finished running. When the slave nodes start, each evolves into an independent subpopulation. All the slave nodes wait for cross nodes to conduct cloning and crossover after the replacement operation. The cross nodes continually check whether all the slave nodes are in waiting state, and if so, update the master population with subpopulations and finish cloning and crossover, then notify all the slave nodes to continue execution. When all the slave nodes finish their specified iterations, the master node completes the final operations of master population updating and selects the optimal antibody. The complete algorithm outline is as follows.

Input data: train set *Train*, validation set *Vali* and test set *Test*.

Parameters: *N*(ranking function number), *T*(iteration number), *β*(clone factor), *M*(processor number).

(1) master node

Initialize master population *P*_*N*_ and partition *P*_*N*_ into *M* subpopulations $P_{N}=\overset{M}{\underset{i=1}{\cup }}S_{i}$. (Each subpopulation corresponds to a unique slave node.)

Master node starts slave nodes and the cross node, then enters a wait state.

Each subpopulation *S*_*i*_ ∈ *P*_*N*_ evolves in parallel and cross nodes run in the background until all the slave nodes finish evolving.

Master node selects the best ranking function from *P*_*N*_ after all the slave nodes finish evolving.

(2) slave node

Initialize subpopulation *S*_*i*_ ∈ *P*_*N*_. Each individual *c* ∈ *S*_*i*_ has a clone pool *Pool*(*c*).

Compute MAP of each ranking function in *S*_*i*_.

For *t* = 1, …, *T*

 Slave node enters a wait state unless the cross node sends a notification.

 Conduct mutation on each ranking function *c*′ in clone pool *Pool*(*c* ∈ *S*_*i*_).

 Compute MAP of each ranking function *c*′ in clone pool *Pool*(*c* ∈ *S*_*i*_); if the MAP of *c*′ is higher than that of *c*, replace *c* with *c*′.

End For

(3) cross node

While learning is ongoing

 If all the slaves enter a wait state

  Update the master population *P*_*N*_ with the latest subpopulations ${\left\{S_{i}\right\}}_{i=1}^{M}$.

  Conduct cloning and crossover.

  Send notification to all slave nodes.

End While

For readers interested in reproducing the experiments reported here, we have released the source code on GitHub at https://github.com/honbaa/RankBCA.git.

## Experiments

### Experiment Setup

We used LETOR3.0 OHSUMED and LETOR4.0 MQ2007 datasets to conduct our verification experiment. The OHSUMED dataset includes 348566 documents, 106 queries, and 16140 query-document pairs and relevance judgments in total. The relevance judgment includes three levels: 2, 1, and 0, respectively representing “relevant”, “possible”, and “not relevant”. To suit the two-value evaluation measure, only “relevant” is considered relevant. The OHSUMED dataset includes 15 features divided into low features and high features: The low features include 10 features and the high features include five features. Each query-document pair includes 45 features in OHSUMED dataset, because the afore-mentioned 15 features are extracted from three fields: title, abstract, and title+abstract. The MQ2007 dataset includes 1700 queries and 69623 query-document pairs and relevance judgments in total. It has the same three-level labeling method as the OHSUMED and includes 14 different features divided into content features, link features, and hybrid features. The content features extract content, anchor text, title, URL, and full five-part documents to form 40 features; the other six features are extracted from the document. Each query-document pair in MQ2007 accordingly includes 46 feature values. Each dataset was subjected to a 5-fold cross validation experiment to avoid over-fitting, and separate experiments were conducted on OHSUMED and MQ2007. The final experimental results were compared with the benchmark algorithms RankBoost, RankSVM, AdaRank, and ListNet.

We used MAP as the affinity function in our experiment, and compared it against benchmarks on MAP, P@1~P@10 and NDCG@1~NDCG@10. The configuration in RankBCA is described in Table 2.

**Table 2:** Algorithm configuration and parameters.

Parameter	Meaning	Value
*H*	Height of antibody tree	7
*T*	Iteration number	60
*β*	Clone factor	0.5
*N*	Size of population	64
*Exp_Num*	Experiment number	10

*T*, *β* and *N* are the parameters in RankBCA, all of which have important effects on the final results. Different parameters have different adaptive value for different datasets. The parameter values in Table 2 were applied specifically to OHSUMED and MQ2007.

### Evaluation Measures and Evaluation Procedure

In order to objectively evaluate the performance of RankBCA, we respectively accounted for its accuracy, speed-up ratio, and convergence rate.

(1) accuracy

The accuracy of a rank-learning algorithm is expressed by the best learned ranking function it identifies. Again, the most common two-value relevance measure is MAP. For the same ${\left\{\left(q_{i},d_{i},y_{i}\right)\right\}}_{i=1}^{m}$, the function with the highest MAP is preferable. The MAP is calculated as follows: S7 and S8 Equations, where *P*@*k*(*q*) is the precision at position *k*, *l*(*k*) is the label at position *k*, 1 is relevant, 0 is not relevant, and *m* is the document size associated with the query. MAP only supports two-value relevance judgment (multi-value relevance can be judged by NDCG.) For the same ${\left\{\left(q_{i},d_{i},y_{i}\right)\right\}}_{i=1}^{m}$ and position *j*, RankBCA returned a better function than the other algorithms, with higher average *NDCG*(*j*) on all queries. The final relevance judgment was calculated as follows: S9 Equation, where *r*(*j*) is the relevance level of documents at position *j* and *n* is the total size of documents in *π*_*i*_.

The three measures above are query-based. The test dataset includes many queries, the corresponding evaluation procedure on which is as follows.

1. Initialize array *P*[10], *NDCG*[10], *AP*[*SIZE*], where *SIZE* is the size of queries in the test data.
2. Obtain a query and documents associated with the query (some lines have the same query id.)
3. Calculate the relevance score of each query-document pair with the optimal rank-learning function.
4. Sort the documents according to the scores obtained from Step 3 to produce a prediction list.
5. On the prediction list from Step 4, calculate P@1~P@10, NDCG@1~NDCG@10, and AP. Accumulate P@1~P@10 to *P*[1]~*P*[10] separately and NDCG@1~NDCG@10 to *NDCG*[1]~*NDCG*[10] separately, and put AP into *AP* array.
6. Repeat Step 2 to Step 5 until all the queries are completed.
7. Calculate average performance as the final evaluation result, that is, each element in *P*[10] or *NDCG*[10] divided by *SIZE* where MAP is calculated via S8 Equation.

In order to maintain the consistency and correctness of the evaluation result and facilitate appropriate comparison with the benchmarks published in LEOTR, we used the standard evaluation tool provided on the official website; the evaluation tool has two versions, LETOR3.0 and LETOR4.0, which are not interchangeable. The two scripts were written in Perl language and named Eval-Score-3.0.pl and Eval-Score-4.0.pl, respectively. They were applied as follows:

perl Eval-Score.pl [test file] [prediction file] [output file] [flag]

Evaluation scripts need a test file and prediction file to complete the evaluation process. The prediction file includes the predicted scores of the documents by the optimal rank-learning function, in which each score occupies a line corresponding to the query-document pair in the test file. The output file is indicated by parameter [output file], which includes evaluation results of MAP, P@1~P@10, and NDCG@1~NDCG@10. The parameter flag is set to 0 to secure an average result. The ActivePerl Perl (5.20.2–64 bit) version was available and thus utilized for the running environment of the scripts–the StrawberryPerl version was not used for our tests.

(2) speed-up ratio

The speed-up ratio is a key parameter for measuring the performance of parallel algorithms, as it precisely reflects the degree of parallelism. The speed-up ratio *S* is defined as follows:S10 Equation, where *T*_1_ represents the time consumed by the serial program executing once, and *T*_*M*_ represents the time consumed by the parallel program executing once. *M* defines the number of processors used for executing the program in practice. The speed-up ratio was measured separately at *M* of 1, 2, 4, and 8 under the same experimental parameters applied to the OHSUMED dataset.

(3) convergence rate

RankBCA is a global search algorithm. In order to measure the convergence rate of its affinity in the learning process, the affinity (MAP) learning curve was plotted on the OHSUMED fold1 dataset.

### Results

(1) accuracy comparison

In addition to the parameters listed in Table 2, the number of processors was set to eight for the comparison experiment; the eight processors were then run in parallel to obtain the final result as the average of 10 valid experiments. (Several experiments were run and averaged to account for the fact that RankBCA is a random algorithm.) The MAP comparison between RankBCA and the benchmarks is shown in Table 3.

**Table 3:** MAP comparison on benchmarks.

Algorithms	MAP(OHSUMED)	MAP(MQ2007)
RankSVM	0.4334	0.4644
RankBoost	0.4411	0.4662
ListNet	0.4457	0.4652
AdaRank-MAP	0.4487	0.4577
RankBCA	0.4601	0.4710

**Fig 5:**
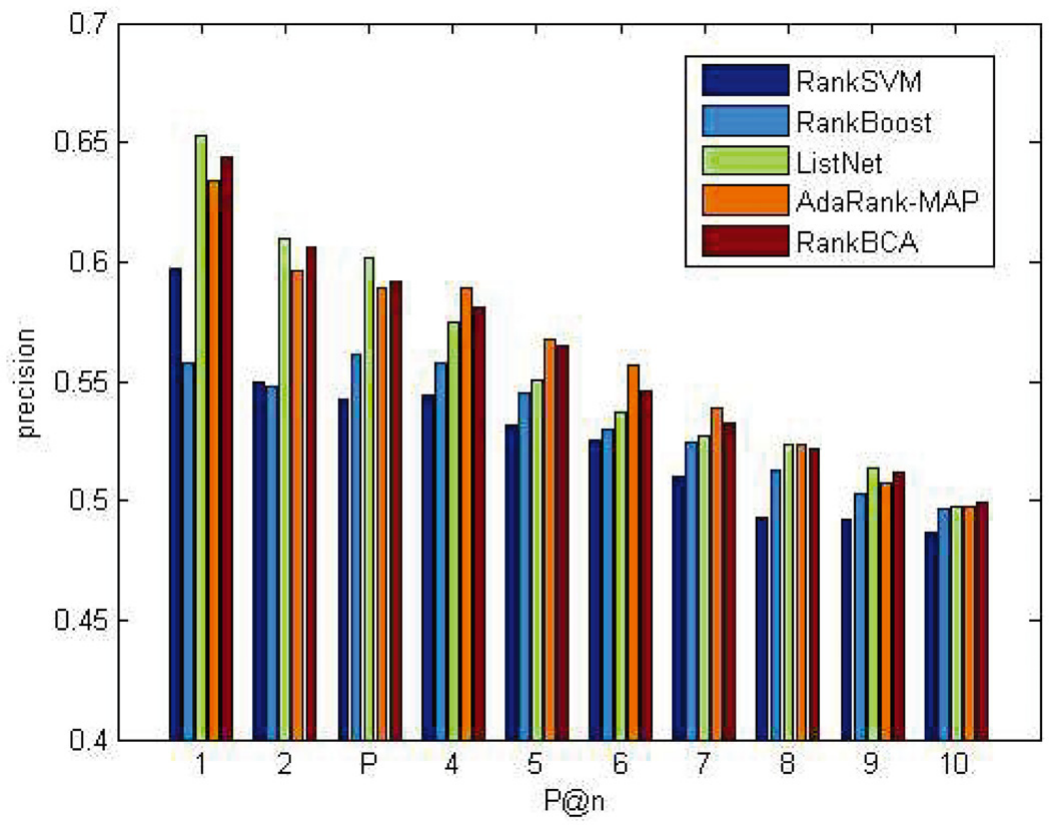
P@n on OHSUMED.

**Fig 6:**
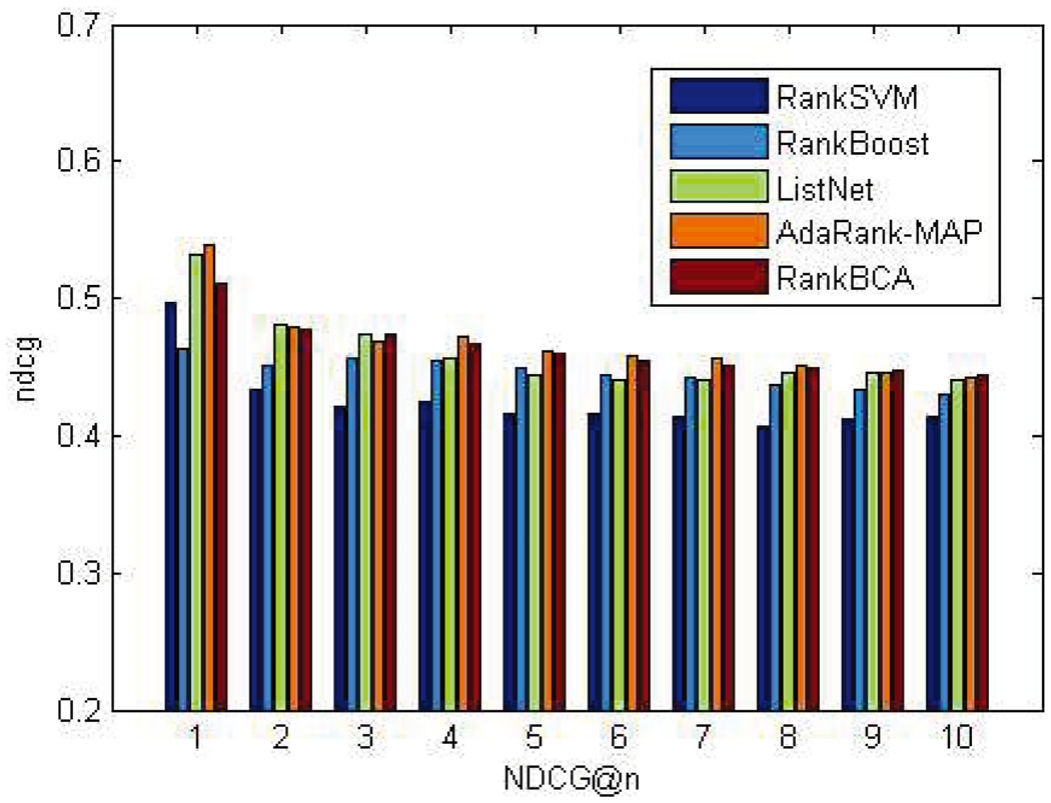
NDCG@n on OHSUMED.

**Fig 7:**
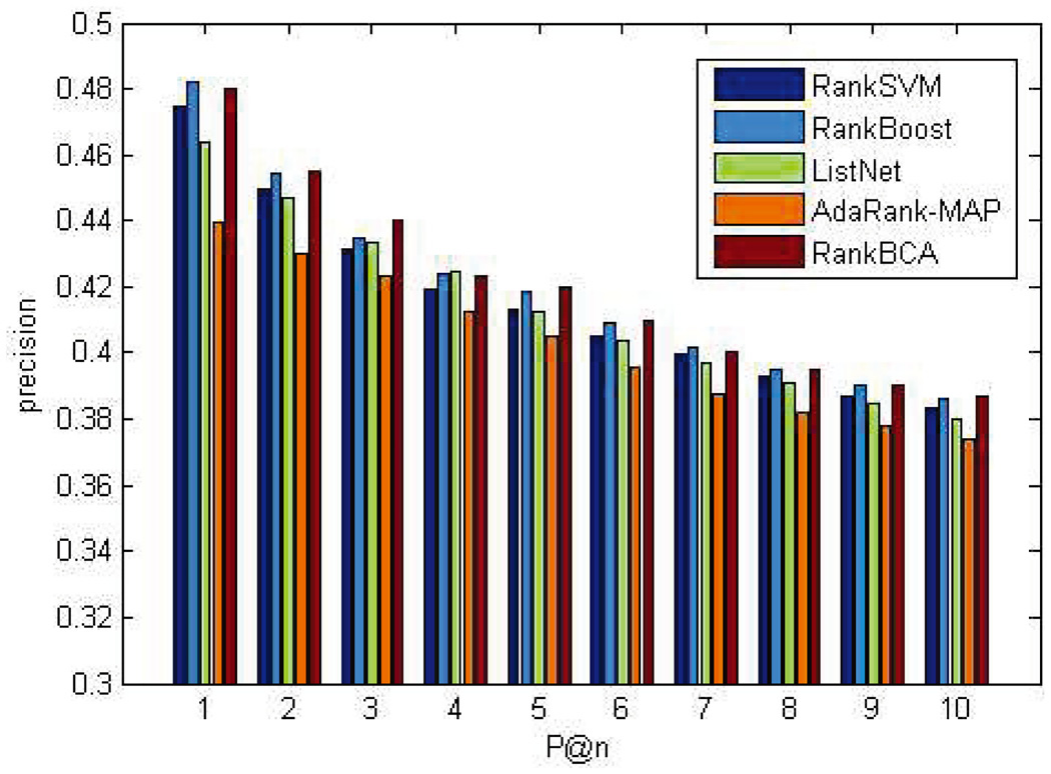
P@n on MQ2007.

**Fig 8:**
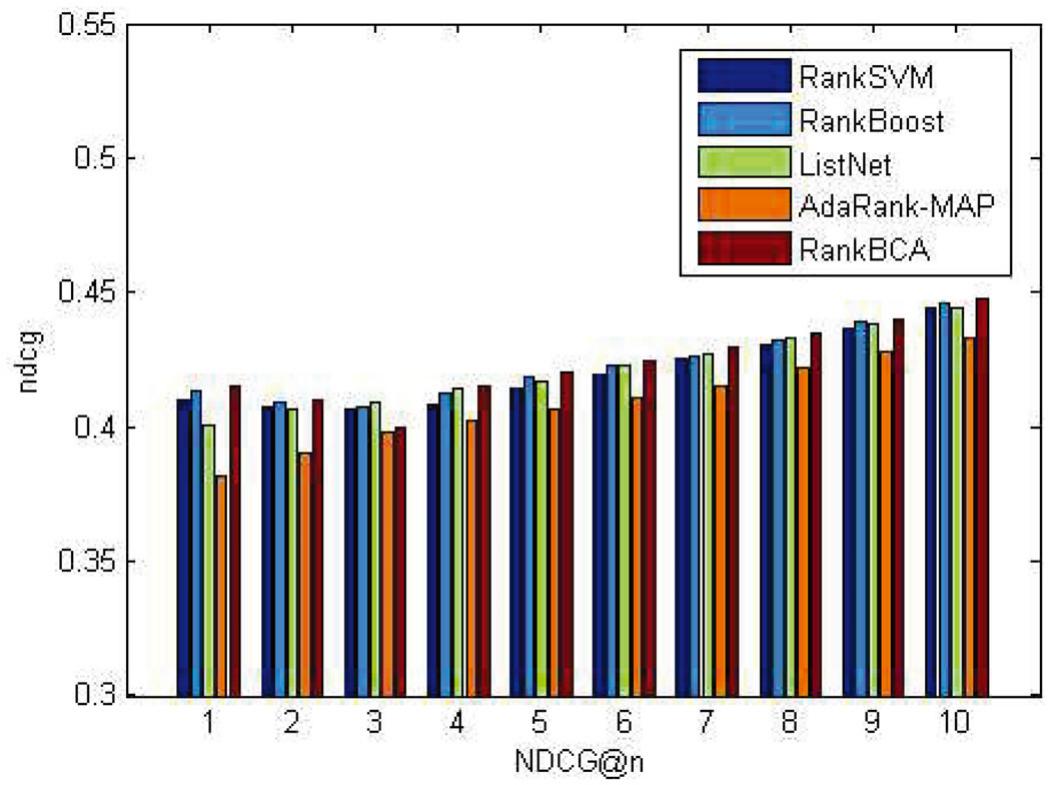
NDCG@n on MQ2007.

P@n and NDCG@n results on the OHSUMED dataset are shown in Figs 5 and 6. P@n and NDCG@n results on the MQ2007 dataset are shown in Figs 7 and 8.

As shown above, regardless of *n* value, the P@n or NDCG@n of RankBCA performed very well compared to the other algorithms (and occasionally outperformed the others.) In respect to precision, RankBCA was more stable than the benchmarks. With the configuration of *M* = 8, RankBCA took 1212 seconds to finish 5-fold validation learning on the OHSUMED dataset. The performance could be improved further if the clone scale increased properly without exerting excessive impact on running time.

(2) convergence rate

**Fig 9:**
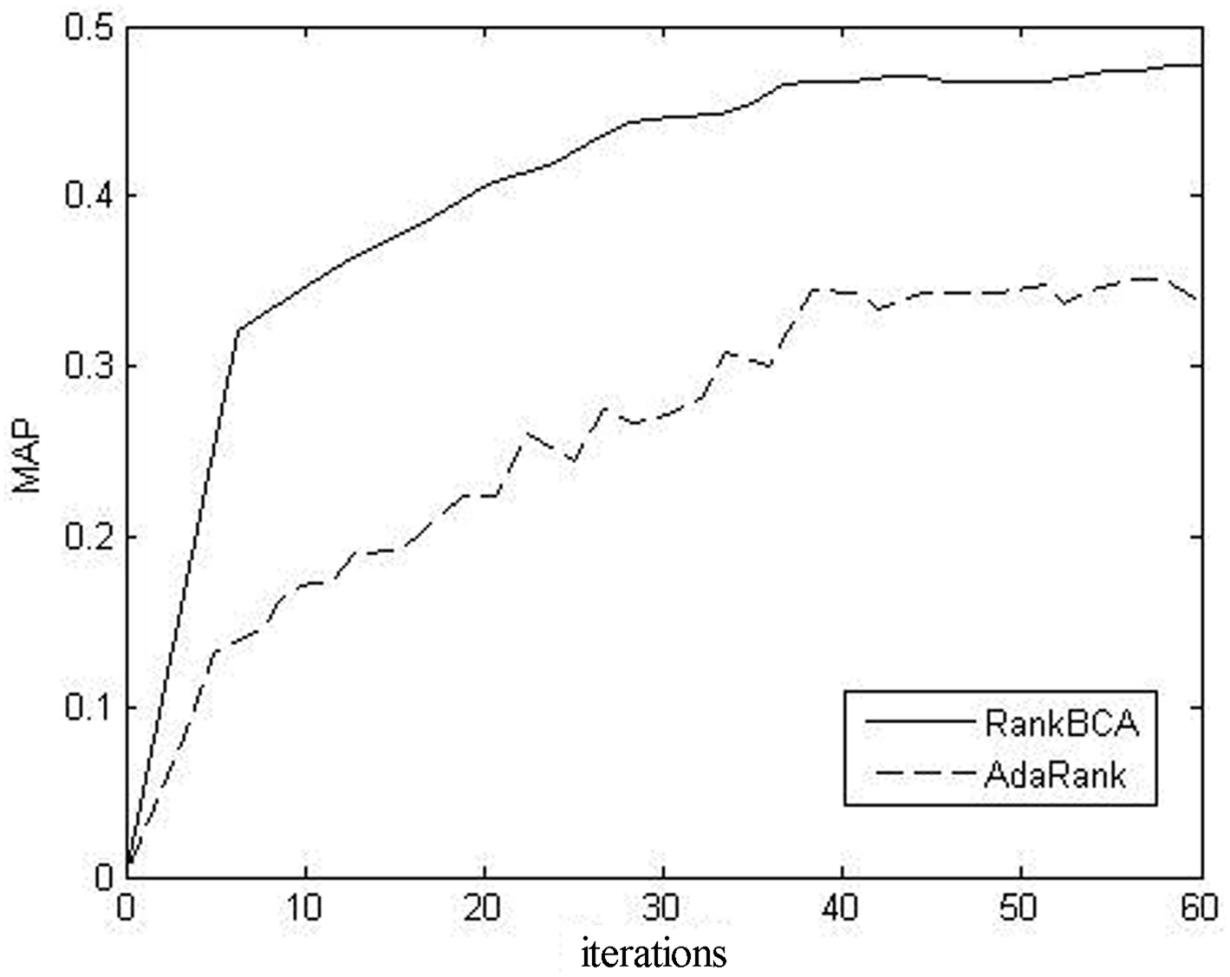
Learning curve of RankBCA.

The learning curve of the MAP for RankBCA on OHSUMED fold1 is shown in Fig 9.

Fig 9 shows that RankBCA tended to be convergent as the iterations progressed, and that its convergence rate was considerably faster than that of AdaRank.

(3) speed-up ratio

The result of the speed-up ratio experiment (again, based on a 5-fold run) is shown in Table 4.

**Table 4:** Speed-up ratio comparison on OHSUMED dataset.

Processor number	Time/s	Speed-up ratio
1	5565	1
2	2914	1.91
4	1661	3.35
8	1212	4.59

Table 4 shows that the time consumed by the proposed parallel algorithm decreased as the processor number increased, while the speed-up ratio increased linearly. The performance of RankBCA was very favorable.

## Conclusion

This paper proposed an innovative parallel BCA designed for rank-learning applications. Compared to similar existing algorithms, RankBCA utilizes population evolution rather than optimizing the loss function to obtain the optimal ranking function. Parallel BCA divides the single population into multiple subpopulations, and then avoids local optima via crossover operation. Each subpopulation occupies an independent processor, which lends very favorable performance. During the evolution procecss, RankBCA utilizes a continuous region mutation on individuals and parallel running to ensure high convergence rate and running speed, while a crossover procedure is applied to the population to enrich its diversity. A comparative experiment confirmed that RankBCA outperforms RankSVM, RankBoost, AdaRank, and ListNet in respect to accuracy and speed on benchmark datasets.

## Supporting Information

S1 Equation (TIF)

S2 Equation (TIF)

S3 Equation (TIF)

S4 Equation (TIF)

S5 Equation (TIF)

S6 Equation (TIF)

S7 Equation (TIF)

S8 Equation (TIF)

S9 Equation (TIF)

S10 Equation (TIF)
