# Supplementary figures and images for: Research on B Cell Algorithm for Learning to Rank Method Based on Parallel Strategy
Source: PLoS One. 2016 Aug 3;11(8):e0157994. doi: 10.1371/journal.pone.0157994 (PMC4972358; doi:10.1371/journal.pone.0157994)

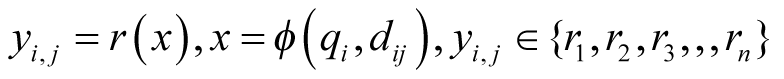

Supplement: S1 Equation — (TIF) [file pone.0157994.s001.tif]

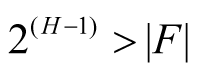

Supplement: S2 Equation — (TIF) [file pone.0157994.s002.tif]

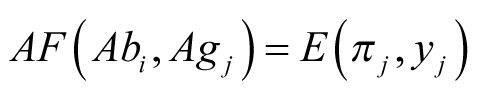

Supplement: S3 Equation — (TIF) [file pone.0157994.s003.tif]

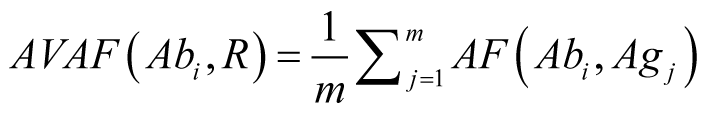

Supplement: S4 Equation — (TIF) [file pone.0157994.s004.tif]

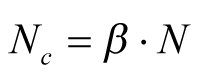

Supplement: S5 Equation — (TIF) [file pone.0157994.s005.tif]

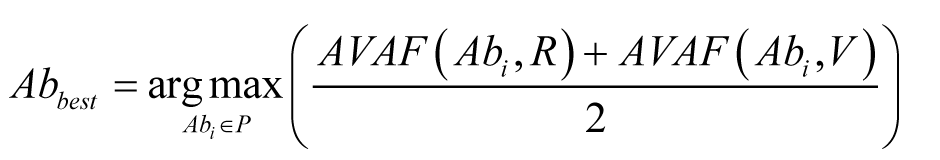

Supplement: S6 Equation — (TIF) [file pone.0157994.s006.tif]

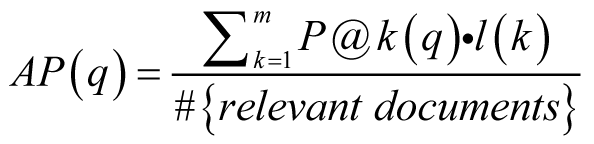

Supplement: S7 Equation — (TIF) [file pone.0157994.s007.tif]

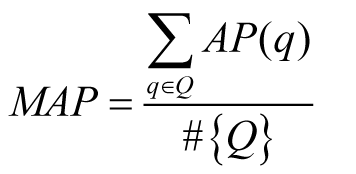

Supplement: S8 Equation — (TIF) [file pone.0157994.s008.tif]

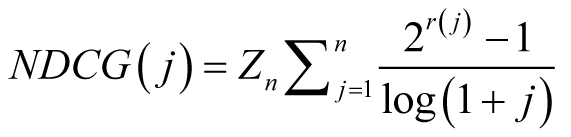

Supplement: S9 Equation — (TIF) [file pone.0157994.s009.tif]

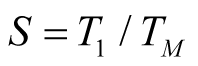

Supplement: S10 Equation — (TIF) [file pone.0157994.s010.tif]
